# Targeting XIST induced apoptosis of human osteosarcoma cells by activation of NF-kB/PUMA signal

**DOI:** 10.1080/21655979.2019.1631104

**Published:** 2019-06-16

**Authors:** Weiliang Gao, Jisheng Gao, Longying Chen, Yande Ren, Jinfeng Ma

**Affiliations:** a Department of Spine Surgery, The 107 Hospital of the People’s Liberation Army, Yantai, Shandong, China; b Department of Orthopedics, Linyi Central Hospital, Linyi, Shangdong, China; c Department of Radiology, The affiliated hospital of Qingdao University, Qingdao, Shandong, China; d Department of Spine Surgery, The affiliated hospital of Qingdao University, Qingdao, Shandong, China

**Keywords:** Osteosarcoma, long noncoding RNA X-inactive specific transcript, nuclear factor-kappa B, p53 upregulated modulator of apoptosis, Apoptosis

## Abstract

The long noncoding RNA X-inactive specific transcript (*XIST*) plays vital roles in tumor progression. However, the underlying mechanisms remain unclear. This study investigated the effects and mechanisms of targeting XIST on osteosarcoma (OS) cells *in vitro* and *in vivo*. We used shRNA to knockdown *XIST* to evaluate cell growth and apoptosis in U2OS cells *in vitro* and xenograft formation *in vivo*. An observed relationship between XIST and the p53 upregulated modulator of apoptosis (PUMA) and nuclear factor-kappa B (NF-kB) pathway was further explored by using small interfering RNA (siRNA). Our results showed that suppression of XIST by short hairpin RNA (shRNA) impeded U2OS cell growth, induced apoptosis and lessened OS xenograft tumor growth. Targeting XIST increased NF-kB-dependent PUMA upregulation in U2OS cells. Upregulation of PUMA is correlated with suppression of XIST-induced apoptosis in U2OS cells. Therefore, inhibition of XIST could promote U2OS cell death via activation of NF-kB/PUMA pathways.

## Graphical Abstract

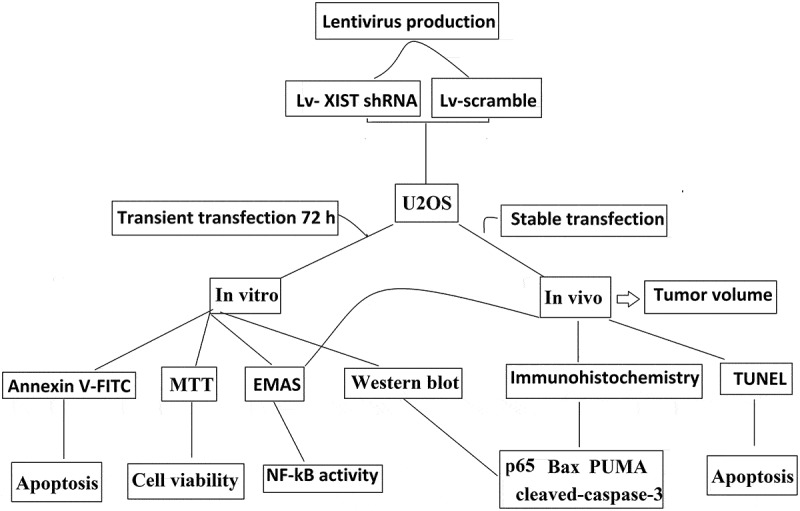

## Introduction

Osteosarcoma (OS) is the most common primary tumor of bone in children and adolescents. It has a poor prognosis due to local recurrence, metastasis, and chemotherapy resistance [1]. Patients without clinical signs of systematic spread show 5-year survival rates of 60–80%, whereas, the 5-year survival for patients with metastatic disease is 20% [2]. Treatment relies on the use of chemotherapy and surgery, but treatment paradigms and survival rates have not improved for the past three decades [3]. Molecular-targeted therapies have been developed recently for the treatment of many cancer types, including OS [4].

Growing evidence demonstrates that long non-coding RNAs (lncRNAs) are involved in the progression of various human tumors [5,6]. These lncRNAs appear to be involved in the regulation of various biological processes, including cell growth, invasion, and apoptosis, as well as cancer progression [7]. The lncRNA X-inactive specific transcript (*XIST*) has been reported to promote multiple cancer progression and was associated with poor prognosis [8].

*XIST* was reported to suppress cell proliferation, metastasis, invasion and exhibited tumor suppressive properties in hepatocellular carcinoma (HCC) [9,10]. However, Mo et al. found that *XIST* promoted cell cycle progression and inhibited apoptosis and exhibited tumor promotive properties in HCC [11]. In OS cells *in vitro* and *in vivo*, targeting XIST inhibited cancer cell proliferation and invasion and suppressed subcutaneous tumor growth in mice [12,13]. However, Zhang et al found that XIST functioned as a tumor suppressor in OS [14]. Therefore, the underlying mechanisms that XIST functions in OS remains unclear.

Recent studies have demonstrated that lncRNAs modulate gene expression by specifically associating either at the promoters or the enhanceing their target genes [15]. The nuclear factor-KB (NF-KB) family of transcription factors play an essential role for the regulation of inflammatory responses, immune function and malignant transformation. Moreover, NF-KB influences the expression of genes that function in cell differentiation, proliferation and survival in almost all multicellular organisms [16]. PUMA (p53-upregulated modulator of apoptosis) is a transcriptional target of the tumor suppressor p53 and a mediator of p53-dependent and p53-independent apoptosis [17,18]. It has recently reported that PUMA mediated TNF-a-induced apoptosis by activating NF-kB pathway *in vitro* and *in vivo* [19]. Pharmacologic inhibition of NF-kB lead to oncolytic protection and significant down regulation of PUMA message levels. And NF-kB activation upregulated PUMA, leading to cell apoptosis, suggesting that PUMA upregulation is dependent on NF-kB activation [20].

Growing evidence suggests that long non-coding RNAs (lncRNAs) have been reported to activate NF-kB signalling through direct interaction between lncRNA and NF-kB or its transcripts, or indirectly through upstream components of lncRNAs [21–24].

In the present study, we explored the role of *XIST* in the treatment of OS. Our results suggest that targeting XIST could inhibit OS proliferation and induced apoptosis in OS cells in vitro and in vivo. We also demonstrated that PUMA is activated by NF-κB in response to shRNA XIST, and that PUMA is a critical mediator of shRNA XIST -induced apoptosis in OS cells *in vitro* and *in vivo*.

## Materials and methods

### Cell culture

The human osteosarcoma U2OS cells were obtained from American Type Culture Collection (ATCC, Shanghai, China), and cultured in McCoy’s 5A, supplemented with 10% heat inactivated FCS 100 U/mL penicillin and 100 μg/mL streptomycin (Invitrogen). Cells were maintained at 37°C in 5% CO2 atmosphere.

### Lentivirus production and infection

Short hairpin RNA targeting XIST (XIST shRNA) or scrambled shRNA were ligated into the LV-3 vector (GenePharma, Shanghai, China). The viruses were packaged in HEK293T cells according to standard protocols, and the packaged lentiviruses were harvested 72 h. The packaged lentiviruses were named Lv- XIST shRNA and Lv-scramble. U2OS cells were transfected with Lv-shRNA and Lv-scramble using Lipofectamine 2000 following the manufacturer’s instructions. To get the stably transfected cells, the cells were exposure to 2 μg/ml puromycin (Sigma, St Louis, MO) for 2 weeks.

### Transfection

To rescue the expression of nuclear factor-kappa B P65 (P65) or PUMA, the stable Lv- XIST shRNA and Lv-scramble cells were co-transfected with P65 siRNA or PUMA siRNA (Dharmacon, Chicago, IL, USA) for 48 h with Lipofectamine 2000 (Invitrogen) following the manufacturer’s instructions.

### RNA extraction and quantitative reverse-transcriptase polymerase chain reaction (qRT-PCR) analyses

Total RNA was isolated using the TRIZOL reagent (Invitrogen, Shanghai, China) from tissues (5–10 mg) and cultured cells according to the manufacturer’s protocol. RNA quality was determined on an Agilent 2100 Bioanalyzer (Agilent Technologies, Guangzhou, China) using the RNA 6000 Nano Assay according to the manufacturer’s protocol. The results were normalized to the expression of Glyceraldehyde-3-phosphate Dehydrogenase (GAPDH). Relative fold change in XIST gene expression were calculated using 2^−ΔΔCt^ method, normalized with respective controls. The primers used in our study were as below: XIST: forward 5'-CTAGCTAGCTTTTGTAGTGAGCTTGCTCCT-3' and reverse 5'-GCTCGATAATGTCATCCTCTCACTTTTGC-3'; GAPDH forward 5'-GCACCGTCAAGGCTGAGAAC-3' and reverse 5'-TGGTGAAGACGCCAGTGGA-3'.

### Western blot analysis

Cells were lysed and centrifuged. Then the samples were separated through a sodium dodecyl sulfate-polyacrylamide gel electrophoresis (SDS-PAGE), transferred to Immobilon P membranes, and western blotting was performed with specific antibodies: p65, PUMA, Bax, cleaved-caspase-3 (Santa Cruz Biotechnology, Santa Cruz, CA) and β-actin (Sigma). Blots are representative of at least three experiments.

### EMSA

Electrophoresis Mobility Shift Assay (EMSA) was performed as reported previously [25]. Briefly, nuclear and cytoplasmic extraction reagents were used to extract the nuclear proteins of U2OS cells and OS tissues. Protein concentration was measured using the BCA Protein Assay Kit (Cat. No. 23227, Thermo Fisher Scientific). The NF-κB probe AGT TGA *G*GG GAC TTT CCC AGG C (Santa Cruz Biotechnology, Shanghai, China) were labeled with [α-^32^P] dCTP, which were incubated with 10 µg nuclear extracts for 30 minutes at room temperature. Anti-p65 antibody (BD Pharmingen) was used to observe a supershift. The reaction mixture was electrophoresed on 4% polyacrylamide gels, and the gel with separated samples were dried and subjected to autoradiography using phosphor screens at −80°C.

### Cell viability and apoptosis assay

Cell viability was examined using the 3-(4, 5-dimethylthiazol-2-yl)-2, 5-diphenyltetrazolium bromide (MTT) colorimetric assay according to the manufacturer’s instruction (Roche, Shanghai, China). Cell apoptosis was detected using the Annexin V-FITC Apoptosis Detection kit (Sigma-Aldrich, St. Louis, MO, USA) according to manufacturer’s instructions. The percentage of apoptotic cells was defined as the sum of early apoptosis (AnnexinV-positive) and late apoptosis (Annexin V-positive and PI-positive) cells.

### Tumorigenesis in nude mice

Animal welfare and experimental procedures complied with national guidelines and were approved by the Animal Experimental Ethical Committee of the central hospital of Linyi, China. Male BALB/c athymic nude mice, 4 to 6 weeks old, were injected subcutaneously with U2OS (2 x 10^7^), stable Lv- XIST shRNA and Lv-scramble/U2OS (2x10^7^) cells. The tumor volume was measured twice weekly for 6 weeks. Tumor size was measured with a digital caliper and calculated using a simple algorithm (3.14× length×wide×height÷6.). After 6 weeks, mice were sacrificed and photographed. Each tumor was fixed and processed for histological examination.

### *Immunohistochemistry and* TUNEL staining

For immunohistochemistry, 4-μm sections of formalin-fixed paraffin-embedded tissue were deparaffinized and rehydrated according to established protocols. Sections were then incubated with anti-PUMA, anti-bax and anti-P65 antibodies (Cell Signaling Technology, Shanghai, China) or phosphate-buffered saline (PBS) at 4°C overnight, followed by sequential incubation with anti-rabbit immunoglobulin secondary antibodies. Samples were visualized by microscopy (I × 81 motorized inverted microscope; Olympus). Cell apoptosis was determined by Terminal deoxynucleotidyl transferase dUTP Nick End Labeling (TUNEL) assay using an ApopTag kit (Millipore; Chemicon International, Temecula, CA) as described previously [26].

### Statistical analysis

Statistical analyses were performed using SPSS 13.0 software (SPSS Inc., Chicago, IL, USA). All experiments were repeated at least three times. Results are presented as means ± SE. Paired Student’s *t*-test was used for statistical analysis, with p < 0.05 considered statistically significant.

## Results

### Targeting XIST inhibits OS cell proliferation and induces cell apoptosis

**Figure 1.**
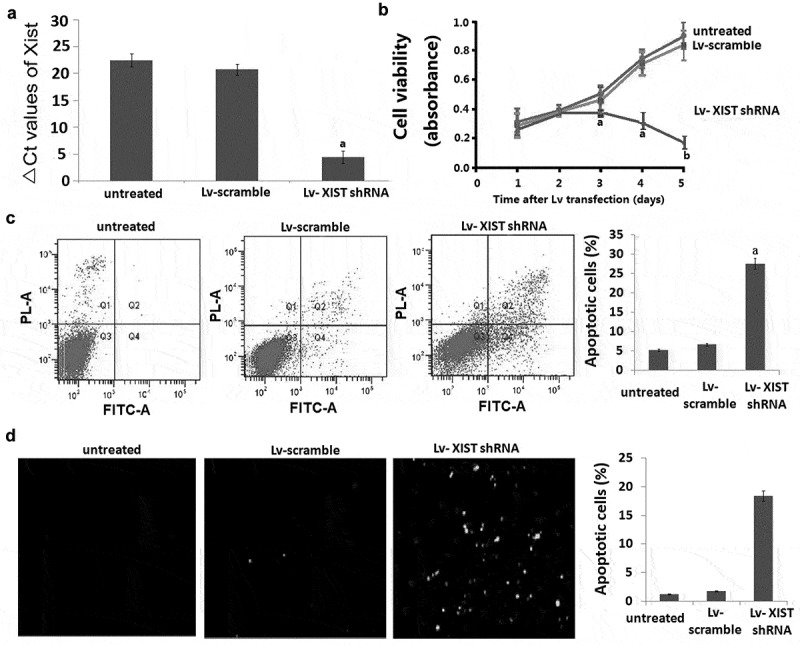
Inhibition of **XIST** by shRNA blocks proliferation and induces apoptosis in U2OS cells. A, U2OS cells were transfected with Lv-shRNA and Lv-scramble for 24 h-96 h. Relative expression of **XIST** in U2OS cells was detected by qRT-PCR. Data were shown using 2^−ΔCT^ values. GAPDH was used for normalization. B, The MTT assay revealed dose-dependent inhibition of cell proliferation after **XIST** shRNA treatment was applied to U2OS cells. C, The results of Annexin V assay combined with propidium iodide assays showed that inhibition of the Xist promoted U2OS cells apoptosis. Data are represented as mean±SD of three independent experiments.D, The results of TUNEL assays showed that inhibition of the Xist promoted U2OS cells apoptosis. Data are represented as mean±SD of three independent experiments.^a^*P*<0.05;^b^*P*<0.01.

**Figure 2.**
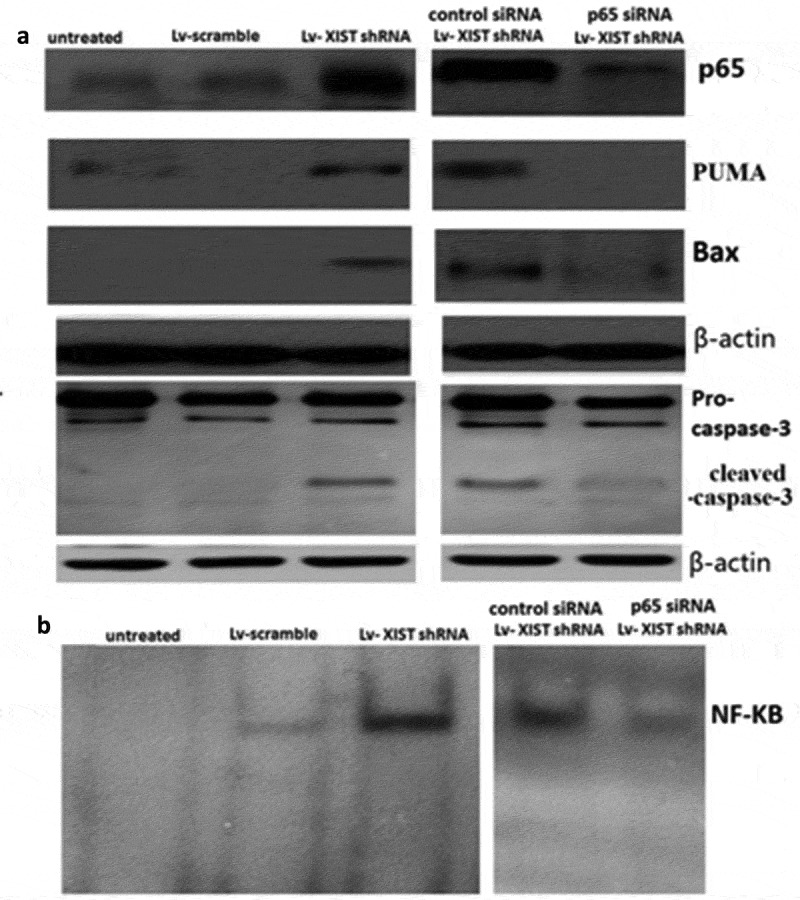
**Targeting XIST induces p65-dependent PUMA expression**. U2OS cells were transfected with Lv-shRNA or Lv-scramble for 72 h, or p65 siRNA (control siRNA) and Lv-XIST shRNA were co-transfected into U2OS cells for 72 h.A, P65,PUMA, bax, cleaved-caspase-3 were detected by Western blot assay; B, NF-KB activity was detected by EMSA assay. β-actin was used for normalization.

Lv- XIST shRNA or Lv-scramble was transiently transfected into U2OS cells for 72 h. The XIST mRNA expression was significantly decreased in U2OS cells by qRT-PCR assay (Figure 1A). As shown in Figure 1B–1D, targeting XIST significantly reduced cell viabilities and induced cell apoptosis of U2OS cells. Cleaved-caspase-3 was also found to be activated in U2OS cell lines by western blot assays after transfection of Lv- XIST shRNA for 72h (Figure 2A).

### Targeting XIST induces p65-dependent PUMA expression

Lv-XIST shRNA or Lv-scramble were transfected into U2OS cells for 72 h. p65 nuclear translocation, PUMA, bax,cleaved-caspase-3 protein expression and the binding activity of NF-Kb was gradually increased in U2OS cells by western blot (Figure 2A) and EMSA (Figure 2B).

To determine whether NF-κB is necessary for the induction of PUMA by XIST downregulation, *p65* siRNA and Lv-XIST shRNA were co-transfected into U2OS cells. With the downregulation of XIST, p65 siRNA abrogated p65 nuclear translocation and PUMA induction by XIST downregulation in U2OS cells (Figure 2A). The binding activity of NF-KB was also blocked (Figure 2B). Moreover, expression of PUMA, bax, cleaved-caspase-3 were downregulated in Lv- XIST shRNA transfected U2OS cells following *p65* siRNA transfection (Figure 2A). The above data collectively indicate that induction of PUMA by XIST downregulation is mediated through NF-κB pathway.

### *Targeting XIST induces PUMA dependent apoptosis in* vitro

**Figure 3.**
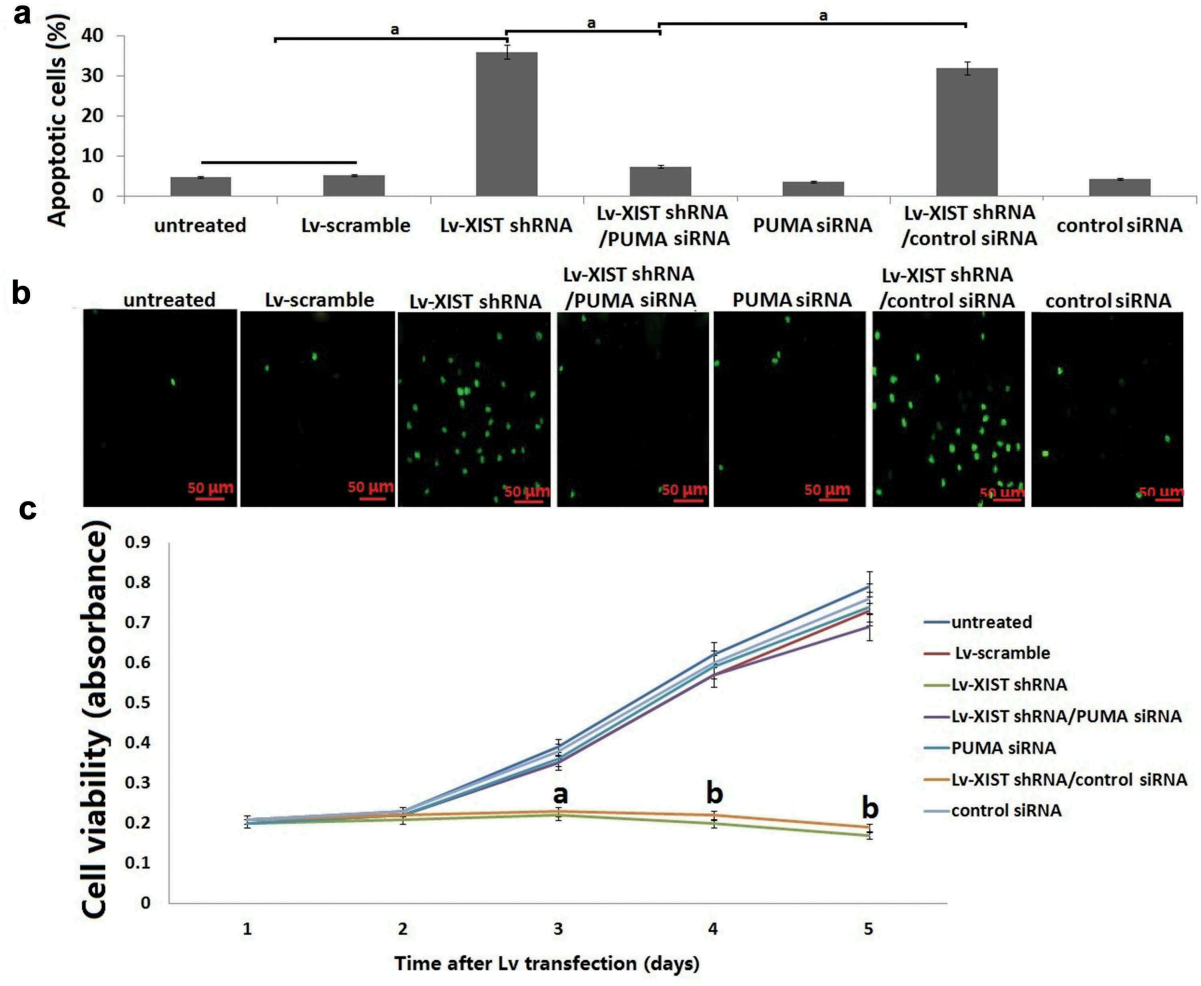
**Targeting XIST inhibits cell viability by PUMA upregulation**. PUMA siRNA and Lv-XIST shRNA were co-transfected into U2OS cells for 96 h. (A) Cell viability in U2OS cells was detected by MTT assay; (B) Cell apoptosis was detected by Annexin V assay combined with propidium iodide assays. (C) Cell apoptosis was detected by TUNEL assays. ^a^*P*<0.05;^b^*P*<0.01.

As shown in Figure 3A, 36% of apoptosis were detected using annexin V/propidium iodide (PI) staining following Lv-XIST shRNA alone for 72 h. However, when PUMA siRNA and Lv-XIST shRNA were co-transfected into U2OS cells for 72 h, Lv-XIST shRNA-induced cell apoptosis was much reduced compared to Lv-XIST shRNA alone. TUNEL assay has the same results as annexin V/propidium iodide (PI) staining (Figure 3B).

### Targeting XIST inhibits cell viability by PUMA upregulation

Cell viability was detected using MTT assay as shown in Figure 3C. Lv-XIST shRNA alone inhibits cell viability, however, co-transfection of PUMA siRNA and Lv-XIST shRNA reversed U2OS cell viability compared to Lv-XIST shRNA alone transfected U2OS cells.

### *Targeting XIST inhibits osteosarcoma growth in in* vivo

**Figure 4.**
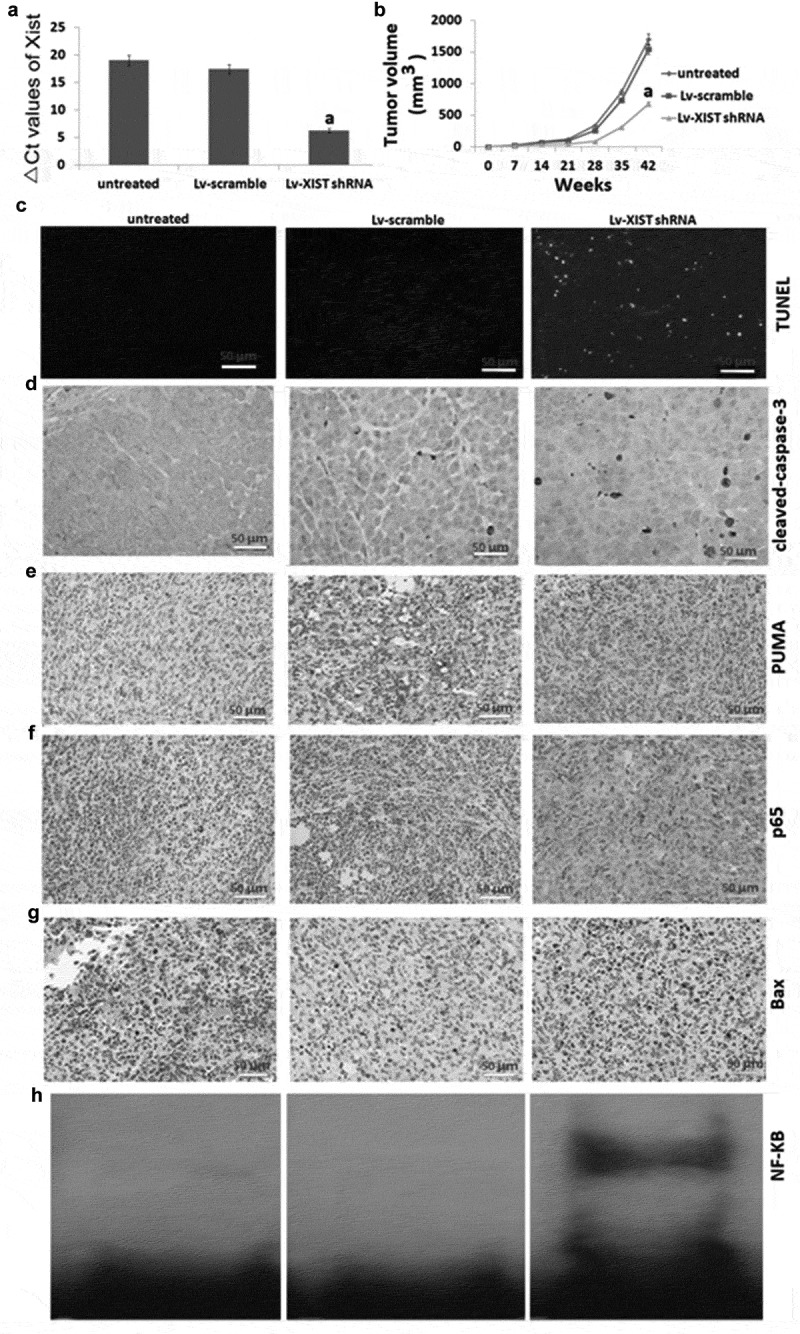
**Targeting XIST inhibits osteosarcoma growth in *in Vivo*, and increases NF-KB activity, P65 nucleus translocation and PUMA expression**. (A) Nude mice received subcutaneous injection of U2OS cells for 7 weeks, relative expression of XIST in U2OS cells was detected by qRT-PCR. (B) Tumor growth curve after nude mice received subcutaneous injection of U2OS cells for 7 weeks. (C) Cell apoptosis by TUNEL assay; Expression of cleaved-caspase-3 (D), PUMA (E), P65 (F), bax (G) in tumors using immunohistochemical staining; H, NF-kB assay was detected by EMSA. ^a^*P*<0.01.

As shown in Figure 4A, in the Lv-XIST shRNA transfected tumor, XIST expression was significantly decreased compared to the Lv-scramble transfected tumors and untreated tumors. Furthermore, the tumors formed by Lv-XIST shRNA transfected U2OS cells grew less and were smaller in size than the tumors formed by Lv-scramble transfected and untreated U2OS cells (Figure 4B). In addition, the tumors established in Lv-scramble transfected U2OS cells showed increased TUNEL-positive cells (Figure 4C) and cleaved-caspase-3 positive cells (Figure 4D). We next analyzed the expression of p65, Bax and PUMA (Figure 4E-4G) expression in the tumor tissues *in vivo*. The results showed that the Lv-XIST shRNA treated U2OS cells demonstrated increased expression of p65, bax and PUMA compared to the Lv-scramble transfected tumors and untreated tumors (Figure 4E-4G). NF-kB was also activated in the Lv-XIST shRNA transfected tumor compared to the Lv-scramble transfected tumors and untreated tumors (Figure 4H).

## Discussion

Long noncoding RNAs (lncRNAs) are commonly defined as non protein-coding transcripts, which have been shown to play crucial roles in cancer biology and correlate with tumorigenesis. The aberrant expression of lncRNAs has been widely observed. Studies have shown that lncRNA was widely involved in both normal physiological processes and pathogenic processes, and played an important role in the pathogenesis of many diseases [27–29].

Reportedly, XIST has been shown to be associated with proliferation, apoptosis, tumor development, metastasis, patient prognosis, and response to chemo- and radio-therapies of cancer. In bladder cancer cells *in vitro*, targeting XIST suppressed cell invasion and proliferation by downregulation of p53 [30]. In nasopharyngeal carcinoma (NPC), targeting XIST inhibited NPC cell proliferation and invasion *in vitro* and NPC tumor growth *in vivo* [31]. Further investigation revealed that XIST acts as an oncogene in non-small cell lung cancer by epigenetically repressing KLF2 expression [32]. These data implicate that XIST drives carcinogenesis by promoting cell proliferation and invasion in cancers. In our study, targeting XIST significantly reduced cell viability and increased cell apoptosis *in vitro* in U2OS cells, and targeting XIST suppressed tumorigenesis *in vivo*. Our findings are similar to those of the recent reports [31,32] and contrary to the results of recent reports [33,34]. However, the underlying mechanism by which XIST mediates gene expression and participates in tumorigenesis remains to be clarified. Xiao et al. has reported that XIST knockdown suppressed the growth of LSCC cells *in vitro* and *in vivo* by modulating the miR-124-EZH2 axis [35]. Yang et al. has reported that XIST enhances OS cancer cell proliferation and invasion in part through the miR-195-5p/YAP pathway [12]. And Li et al. has reported that XIST can promote TGF-β-induced EMT and cell invasion and metastasis by regulating miR-367/miR-141-ZEB2 axis in NSCLC [36]. Therefore, the signaling pathways by which XIST plays in different tissues and cells are different.

It is well known that constitutive activation of NF‐*κ*B pathway contribute to cancer development and persistent tumor survival by activation of downstream anti-apoptotic pathways [16]. However, it has recently reported that TNF-αor mitotic arrest imposed by aurora kinase inhibition could induce cell apoptosis by activation of NF-κB and NF-κB-dependent PUMA upregulation [19,37], suggesting the activation of NF-KB has the pro-apoptotic role by upregulation of PUMA signal. lncRNAs have also been demonstrated to activate NF-kB signalling through direct interaction between lncRNA and NF-kB or its transcripts, or through upstream components [21–24]. It has recently found that the expression of XIST was promoted by activated NF-κB pathway and, in turn, XIST generated a negative feedback loop to regulate NF-κB/NLRP3 inflammasome pathway for mediating the process of inflammation [38]. Zhao et al. also reported that targeting XIST inhibited NF-kB activity, which serving as a therapeutic target in neuropathic pain [39]. In our study, we found that targeting XIST results in constitutive activation of NF‐*κ*B and p65 nucleus translocation and PUMA signal pathway, whereas P65 inhibition could attenuate the PUMA signal expression in the XIST shRNA transfected U2OS cells, demonstrating that XIST could modulate PUMA signaling through activation of P65. In addition, targeting PUMA inhibits apoptosis and promotes cell viability in the XIST shRNA transfected U2OS cells, suggesting that targeting XIST inhibits cell viability and induces apoptosis via P65 dependent PUMA signal pathway. Our results revealed the regulatory network among XIST, P65 and PUMA in OS cells, and confirmed the key role of PUMA in XIST downregulation-induced anti-tumor effect.

## Conclusion

Targeting XIST suppressed cell proliferation and tumorigenesis through activation of NF-kB and NF-kB-dependent PUMA signal. These findings suggested that XIST may serve as a potential therapeutic target for OS treatment.

## Highlights

1. Targeting XIST inhibits proliferation and induces apoptosis in U2OS cells *in vitro*

2. Targeting XIST inhibits tumorigenesis *in vivo*

Targeting XIST inhibits proliferation and induces apoptosis by p65-dependent PUMA signal
